# Rare Complete Restoration of Intraepidermal Nerve Fibre Density by Immunoglobulins in Post-COVID Vaccination Syndrome-Associated Small Fibre Neuropathy: A Case Report and Literature Review

**DOI:** 10.7759/cureus.114178

**Published:** 2026-08-08

**Authors:** Vanessa Schmidt-Krüger, Josef Finsterer

**Affiliations:** 1 Molecular Biology, Inmodia GmbH, Institute for Molecular Diagnostics, Passau, DEU; 2 Neurology, Neurology and Neurophysiology Center, Vienna, AUT

**Keywords:** intraepidermal nerve fibre density, post-acute covid vaccination syndrome, skin biopsy, small fibre neuropathy, spike protein

## Abstract

This report describes the rare complete restoration of reduced intraepidermal nerve fibre density (IENFD) following the administration of intravenous immunoglobulins (IVIG) in a patient with post-acute COVID-19 vaccination syndrome (PACVS) that manifested, among other symptoms, as small-fibre neuropathy (SFN).

The patient was a 53-year-old woman who developed SFN in June 2021 following her second BNT162b2 vaccination. Clinically, the SFN presented with symptoms including sensory disturbances, arterial hypotension, and orthostatic tachycardia. After eight cycles of IVIG therapy from June 2024 to February 2025, three years after the onset of PACVS, a significant improvement in the clinical SFN symptoms was observed. Additionally, the IENFD improved from 6 at the ankle and thigh before IVIG therapy to 9 at the ankle and 8 at the thigh after therapy. The skin biopsy in 2026 was unremarkable. The spike protein was detectable in peripheral blood mononuclear cells, as were vaccine plasmid DNA remnants in the skin biopsies before and after IVIG therapy. S100 protein staining was weakly positive before and after IVIG administration.

We report the case of a 53-year-old female patient who presented with sensory and autonomic disturbances; following a skin biopsy, she was diagnosed with PACVS-associated SFN. Treatment with IVIG over several months led to an improvement in symptoms and a complete restoration of IENFD. This case highlights the importance of diagnosing PACVS-associated SFN and the beneficial effect of IVIG, which not only improves the clinical manifestations of SFN but also normalises IENFD.

## Introduction

SARS-CoV-2 vaccinations are generally well tolerated but can cause various adverse reactions in individual patients [1]. These adverse reactions manifest either as acute COVID-19 vaccine-associated syndrome (ACVS) within hours or a few days after vaccination or as post-acute COVID-19 vaccine-associated syndrome (PACVS) several days, weeks, or months after vaccination [2]. Despite increasing knowledge about their pathophysiology, ACVS and PACVS remain evolving and debated clinical entities. One of the acute adverse reactions in ACVS is, for example, shoulder injury related to vaccine administration (SIRVA) [3]. Another system frequently affected by ACVS or PACVS is the peripheral nervous system (PNS) [1]. One of the PACVS manifestations in the PNS is small fibre neuropathy (SFN), which presents as sensory disturbances, focal pain or regional pain syndrome, dysautonomia, and a reduction in intraepidermal nerve fibre density (IENFD) [4]. The IENFD counts the tiny sensory nerve fibres in the outer layer of the skin and thus serves as a measure used to diagnose SFN. SARS-CoV-2 vaccination-associated SFN is rarely reported, and there are no established therapies, but most patients receive symptomatic treatment, immunosuppressive therapy, or intravenous immunoglobulins (IVIG). However, there is limited evidence supporting the benefit of these measures [5]. The following case is noteworthy for PACVS-associated SFN that responded favourably to IVIG, highlighting the need for heightened awareness of this condition among clinicians.

## Case presentation

A 53-year-old woman with a past medical history of symphysial arthrosis and migraine developed ACVS immediately after her second BNT162b2 vaccination in June 2021, which completely resolved after a few days. One month later (July 2021), she complained of fatigue and orthostasis [6]. Two months later, in September 2021, she developed exercise intolerance, nausea, loss of appetite, exertional dyspnoea, shortness of breath, anginal chest pain, palpitations, arterial hypotension, hoarseness, dysarthria, muscle tension, impaired concentration, brain fog, memory impairment, oversensitivity to light, and insomnia. PACVS was suspected. These symptoms significantly impacted activities of daily living. In November 2021 carpopedal spasms developed [6]. In December 2021 right facial palsy was diagnosed. In January 2022 she recognised sore lymph nodes for the first time. In February 2022 sensory disturbances of both hands became apparent. In March 2022 postural orthostatic tachycardia syndrome (POTS) was diagnosed [6]. Since April 2022, she has developed chewing difficulty and recurrently observed fasciculations of the lower limb muscles. Since June 2022, she has also recognised heat intolerance and sensory disturbances in both feet [6, 7]. In January 2023, protein S deficiency was detected. A skin biopsy in August 2023 revealed reduced IENFD (6/mm at the ankle and thigh (reference values according to Bakkers et al. [8])). In October 2023, chronic myopericarditis was diagnosed. Also, in October 2023, SFN was diagnosed based on the clinical presentation (hypoesthesia, paraesthesia, pallhypoaesthesia, hypohidrosis, orthostatic intolerance, palpitations, POTS, presyncope, decreased baroreceptor sensitivity, blood pressure variability, delayed bladder emptying, and insomnia (Table 1)), quantitative sensory testing, and the abnormal skin biopsy [6, 7]. Despite treatment trials with glucocorticoids, heparin-induced extracorporeal low-density lipoprotein (LDL), fibrinogen precipitation (HELP), and ivabradine (Table 2), the SFN did not regress, and the clinical manifestations persisted.

**Table 1:** Changes of symptoms after intravenous immunoglobulin therapy AA: almost absent, CNS: central nervous system, I: improvement, NI: no improvement, PNS: peripheral nervous system, POTS: postural tachycardia syndrome, 1 weight gain 4 kg, RR: blood pressure

Symptoms present in 08/2023	Symptom change in 02/2025
General fatigue	I
Exercise intolerance	I
Postexertional malaise	I
Nausea	AA
Loss of appetite 1	AA
Sore lymph nodes	I
Rhinoconjunctivitis	AA
Cardiovascular exertional dyspnoea	I
Pressure on chest	AA
Shortness of breath	AA
Anginal chest pain	I
Arterial hypotension	NI
Extensive RR variability	NI
PNS orthostasis	I
POTS	I
Presyncope	I
Hoarseness	I
Dysarthria	AA
Paraesthesias of tongue and mouth	AA
Right facial palsy	AA
Numbness right face	AA
Ptosis	I
Chewing weakness	AA
Paraesthesias of neck	AA
Hypertense axial muscles	I
Hypertense respiratory muscles	I
Fasciculations (legs)	AA
Weakness of hands	I
Hypoesthesia of hands	I
Hypoesthesia/ paraesthesias of feet	I
Heat intolerance	I
CNS Headache	I
Impaired concentration	I (still occurs with overload)
Brain fog	I (still occurs with overload)
Memory impairment	I (still occurs with overload)
Loss of multitasking ability	I
Oversensitivity to light and noise	I
Sleep disorder	I

**Table 2:** Non-specific treatment of the patient for SFN and other indications since 10/2023 + fair, ++ very good, O no effect ME/CFS: myalgic encephalitis/chronic fatigue syndrome, POTS: postural tachycardia syndrome; EBV: Epstein–Barr virus; CMV: cytomegalovirus; VZV: varicella-zoster virus; TSH: thyroid-stimulating hormone; IVIG: intravenous immunoglobulin; SFN: small-fibre neuropathy; d: days

Date	Therapy	Indication	Effect
Since August 2023	Naltrexone 4.5 mg/d	Off-label ME/CFS	+
Since August 2023	Rosuvastatin 15 mg/d	Vasoprotection	O
Since August 2023	Apixaban 5 mg/d	Hypercoagulable state	++
Since August 2023	Clopidogrel 75 mg/d	Hypercoagulable state	++
Since August 2023	Cholecalciferol 1000 IE/d	Low vitamin D levels	O
Since August 2023	Zinc 25 mg/d	Low zinc levels	O
Since August 2023	Copper 3 mg/d	Low copper levels	O
Since October 2023	Ivabradine 5-10 mg/d	POTS	+
Since November 2023	Prednisolon 5 mg/d	Suspected myopericarditis	+
November 2023 to October 2024	Losartan 25 mg/d	Myopericarditis	O
Since November 2023	Magnesium 300 mg/d	Prevention of arrhythmias	O
In November 2023	Ganciclovir infusions 1x500 mg/d	Possible activation of EBV, CMV, VZV	+
Since November 2023	Valaciclovir 1500 mg later 500 mg; Inosin/dimepranol-4-acetamidobenzoat 1500 mg; Melatonin 2 mg	Possible activation of EBV, CMV, VZV	+ O +
Since November 2023	Nattokinase 6000 FU	Thrombolysis	O
January 2024 to April 2024	Sulodexid 250 ULS; N-Acetylcystein 1800 mg; Ginkgo biloba 24 mg	Fibrinolysis, thrombolysis	O O O
Since March 2024	Ketotifen 2 mg/d; Famotidin 40 mg/d; Rupatadin 10 mg/d; Pycnogenol 200 mg/d; Alpha-lipoic acid 450 mg/d	Increased histamine and IgE, mast cell stabilisation	+ + + O O
Since April 2024	Low-dose aripiprazole, up to 2 mg/d	Neuroinflammation	+
Since August 2024	NaCl infusions 0.9 %, 1 l initially daily, later 1-2x week	POTS	++
Since October 2024	Levothyroxin/potassium iodide 75 µg/98.1 µg	Increased TSH	+
Since November 2024	Sacubitril/valsartan 24 mg/26 mg	Incipient heart failure	O
Since June 2024: 90 g for 4 d (June 2024), 90 g for 3 d (August 2024), 90 g for 3 d (September 2024), 55 g for 2 d (October 2024), 60 g for 2 d (November 2024), 60 g for 2 d (December 2024), 40 g for 1 d (January 2025), and 40 g for 1 d (February 2025).	IVIG	SFN	++

Between June 2024 and February 2025, the patient received eight cycles of IVIG, resulting in a significant improvement in several clinical symptoms of SFN (Table 1). SFN symptoms that almost completely resolved included tongue paraesthesia, right-sided facial paralysis, neck paraesthesia, and others (Table 1). Orthostatic dysplasia, presyncope, hypoesthesia, heat intolerance, and muscle hypertonia also improved after IVIG administration (Table 1). IENFD in skin biopsies taken from the ankle and thigh in February 2025 had improved to 9/mm (ankle) and 8/mm (thigh), respectively, after eight IVIG cycles. A third skin biopsy from 2026, in which IENFD was assessed according to the Lauria criteria, showed normal results.

**Figure 1:**
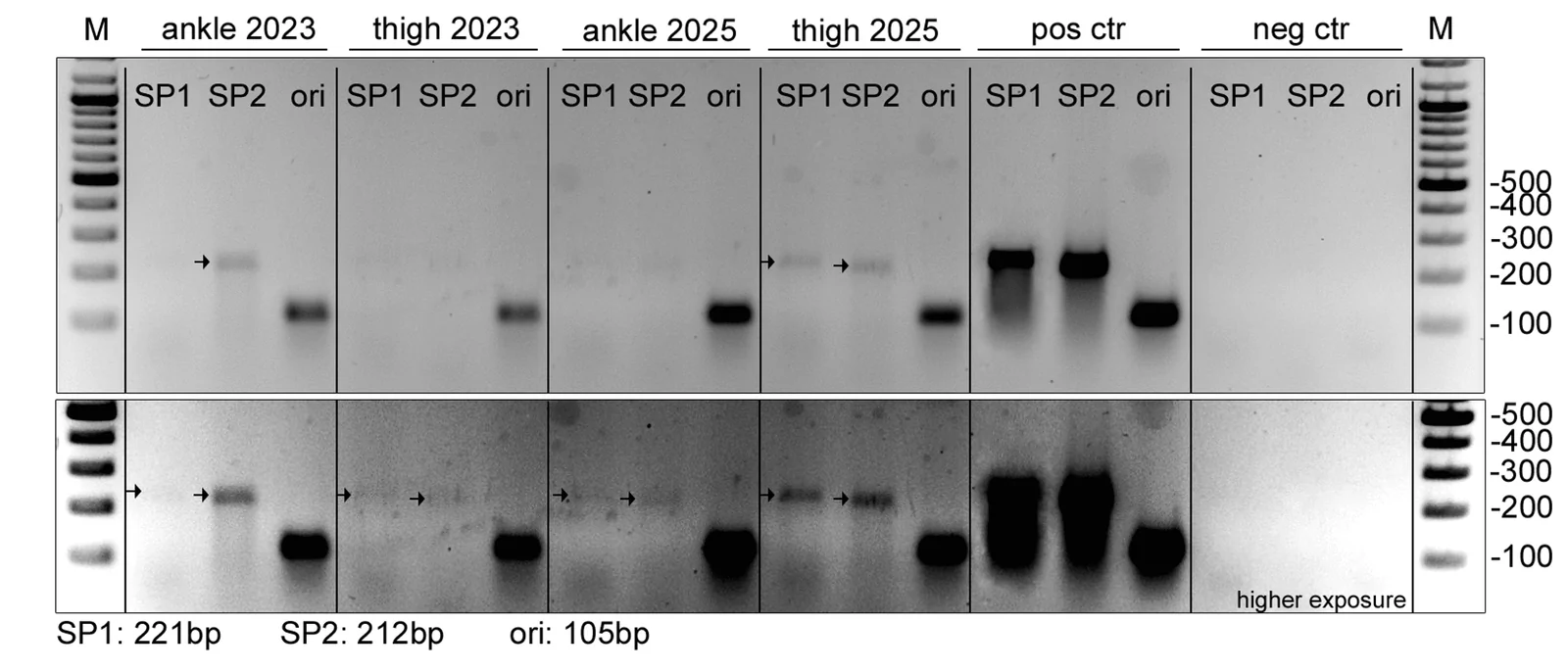
Detection of plasmid DNA sequences in skin biopsies. Qualitative detection of two different spike protein sequences in isolated total DNA after agarose gel electrophoresis of skin biopsies from 2023 and 2025. The arrows mark the bands representing the different plasmid sequences. The ori sequence served as a control for DNA integrity. SP: spike protein; ori: origin of replication; bp: base pairs; M: marker; pos ctr: positive control (vaccine vial); neg ctr: negative control (without template).

Spike protein measurements in serum, exosomes (extracellular vesicles), and peripheral blood mononuclear cells (PBMCs) (B-lymphocytes, T-lymphocytes, NK-cells, and monocytes) in 2023 prior to IVIG administration showed the presence of spike protein in PBMCs but not in serum or exosomes. Spike protein measurements in July 2025 again yielded negative results in serum and exosomes; however, spike protein was detected in PBMCs at a titre of 17.5 fg spike protein/1 mg total protein. Molecular analysis of skin biopsies from 2023 and 2025 identified two independent spike protein sequences in the residual plasmid DNA (SP1 and SP2) of the BNT162b2 vaccine (Figure 1, Appendix A). The ori sequence served as proof of the integrity of the isolated DNA and could have originated either from the plasmid DNA or from the immune cells that presumably eliminated not only the vaccine itself but also host bacteria. No SV40 sequence carrying the vaccine could be detected. The spike protein itself was no longer detectable in the tissues examined.

**Figure 2:**
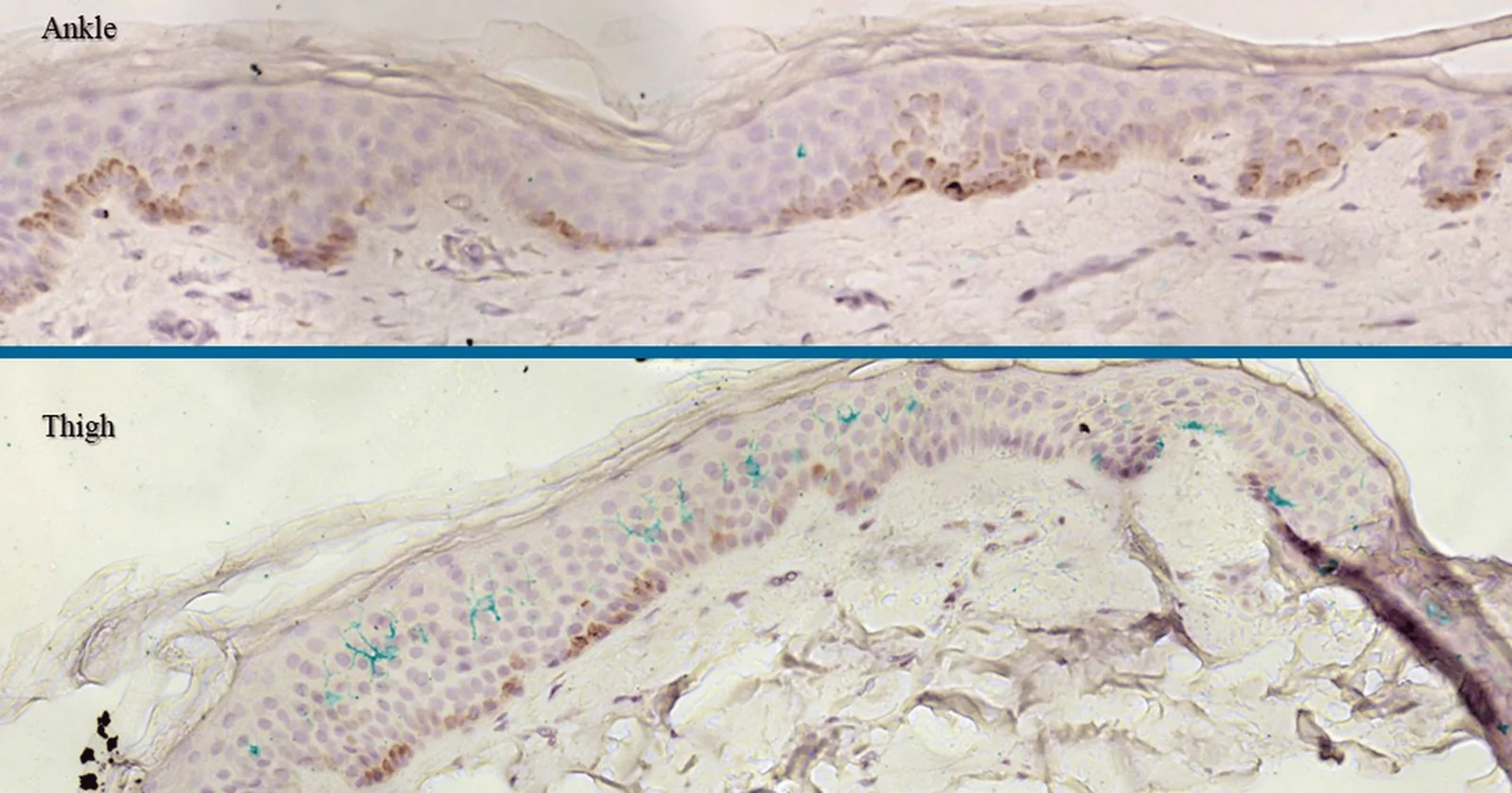
Qualitative S-100 staining in skin biopsies. S-100-positive cells in the epidermis of skin tissue (ankle and thigh) from the year 2025 are shown in green. The cell nuclei were counterstained with haematoxylin (violet). Scale bar: 50 µm.

Immunohistochemical analysis of skin biopsies from 2023 for S100 proteins showed weakly positive to no staining in the ankle and thigh biopsies. The ankle biopsy from 2025 also showed weakly positive to no staining (Figure 2). In contrast, the thigh biopsy from 2025 showed a strongly positive reaction for S100 proteins with clearly visible fibres. Molecular biological analysis of the skin biopsy from 2023 detected the spike sequence fragment-1 and the origin of replication (ORI) sequence but was negative for the SV40 sequence in both the ankle and thigh biopsies.

## Discussion

The patient presented in this case report is interesting for several reasons. First, she is one of the few in whom the symptoms of SFN improved not only clinically after administration of IVIG but also with regard to IENFD. Previous reports of a positive effect of IVIG on IENFD include the case of a 39-year-old man from the USA who developed SFN after the first dose of BNT162b [9]. He presented clinically with increasing pain, burning dysaesthesia, and numbness, initially only in his left arm, later generalised [9]. Nerve conduction velocity testing was normal, but skin biopsies of the left calf and left foot revealed an IENFD of 2.95/mm (normal: >4) in the left calf and 1.83/mm (normal: >3) in the left foot [9]. Additionally, the sweat gland nerve fibre density (SGNFD) was reduced to 7.4 (normal >30/mm) in the left distal arm and to 3.0 (normal 36.5/mm) in the left calf [9]. The patient also exhibited high positivity for FGFR3 antibodies. He benefited from IVIG administration, not only in terms of his sensory disturbances and pain, but also with regard to IENFD, which increased to 5.6 (foot) and 8 (calf) 405 days after SC2V implantation [9]. In contrast, a study of seven patients with SC2V-associated SFN and arthropathy who received only steroids and immunosuppressants, but no IVIG, showed no significant change in IENFD after 12 months, although the clinical condition of these seven patients improved [10]. In an observational study of 23 patients with PACVS-associated SFN, in whom skin biopsy was diagnostic in 52% of cases, three patients did not recover after nine months of steroid therapy [11]. However, after administration of IVIGs, the SFN symptoms resolved in all three patients within two weeks [11]. Unfortunately, a second skin biopsy was not performed in these patients after IVIG treatment [11]. Whether the clinical improvement or the positive biopsy findings in all of these previously described patients and the index patient are actually attributable to the IVIGs remains speculative, as spontaneous remission of clinical symptoms and findings, as well as recovery of the SFN and IENFD independently of the IVIGs, is also conceivable. However, a positive effect of the IVIGs is likely in the index patient, since other prior treatments showed no effect. Differential causes of SFN that were excluded in the index patient include primary (genetic) SFN and secondary (acquired) SFN, SFN due to diabetes, vitamin B12 deficiency, copper deficiency, hypothyroidism, renal insufficiency, Sjögren syndrome, sarcoidosis, coeliac disease, systemic lupus erythematosus, viral infections, heavy metal exposure, alcohol abuse, antiretrovirals, chemotherapeutics, and paraneoplasia. Possible alternative explanations for the improvement in SFN, in addition to IVIG treatment, include a cumulative effect of previously administered therapies or the natural course of PACVS.

The second interesting point is that PACVS was confirmed by the detection of spike protein in PBMCs. Spike proteins are produced by various body cells following SC2V and the uptake of lipid nanoparticles (e.g., Pfizer and Moderna) or vector vaccines (e.g., Janssen and AstraZeneca) [12]. Spike proteins are cleaved from the cell surface and released into the bloodstream as free spike proteins or via exosomes. The absence of detectable spike proteins in plasma could also be explained by limitations of the assay (it only measures free, antibody-unbound spike proteins) and/or by the rapid clearance of antibody-bound spike proteins through complement activation. Simultaneously, PBMCs also take up the vaccine and express spike proteins. Monocytes and macrophages, subpopulations of PBMCs, circulate throughout the body, where they recognise and eliminate vaccine-transfected, spike protein-producing cells. Therefore, spike protein remnants can subsequently be detected in immune cells. More importantly, however, monocytes and macrophages may exhibit intracellular persistence of the spike protein [12]. The latter is frequently associated with “spikeopathy” or PACVS, in which the spike protein remains stored in specific immune cells (e.g., CD16+ monocytes) for months [12]. The negative serum/exosome result indicates a decrease in the active, systemic circulation of the protein, while PBMC positivity points to an intracellular protein reservoir. The intracellular presence of the spike protein in PBMCs may maintain chronic inflammation and contribute to persistent symptoms such as cognitive impairment, fatigue, and vascular dysfunction.

The third interesting point is the detection of the S100 protein in skin biopsies. S100 proteins serve as biomarkers for persistent immune system dysregulation, inflammation, and cellular stress following vaccination [13]. They are typically released by damaged or activated cells and act as alarmins, maintaining chronic inflammation. The reappearance of S100 proteins in the thigh skin biopsy after IVIG administration suggests that chronic inflammation persists in the index patient [13]. The findings in the index patient are consistent with previous case reports showing that vaccine mRNA, plasmid DNA, spike protein, and genomic dysregulation can persist for at least 3.5 years after SARS-CoV-2 vaccination [14].

A fourth point of interest is that retrospective analyses of skin biopsies from 2023 revealed the presence of two independent spike sequences and possibly the ORI sequence but the absence of the SV40 sequence in the ankle and thigh regions, a sequence that was still detectable in 2025. This pattern suggests that remnants of bacterial plasmid DNA (e.g., pBR322), used in the BNT162b2 manufacturing process, were present in the tissue [13]. The spike sequences correspond to the codon-optimised DNA sequence of the SARS-CoV-2 spike protein that was inserted into the vaccine construct. The DNA test does not detect the sequence of the viral spike protein itself. The ORI sequence indicates either the presence of the bacterial backbone (e.g., pBR322), required for DNA plasmid replication in *Escherichia coli* during manufacturing, or originates from immune cells that presumably eliminated the host bacteria alongside the vaccine itself. The absence of the SV40 sequence suggests that the SV40 promoter-enhancer sequence (used to enhance expression in eukaryotic cells) is either absent or below the detection limit of the specific PCR test [14].

## Conclusions

In summary, this case aligns with previous reports about PACVS-associated SFN. However, it is unique in the improvement of clinical manifestations of SFN and restoration of IENFD following IVIG administration. The case underscores two learning points: 1. The spike protein persists in PBMCs while simultaneously absent in serum and exosomes, which is consistent with PACVS; 2. Immune dysregulation following SARS-CoV-2 vaccination persists despite clinical improvement after IVIG administration. This case highlights the importance of diagnosing PACVS-associated SFN and the beneficial effect of IVIG.

## Appendices

Appendix A

Methods

DNA extraction and gene expression from tissue: Formalin-fixed tissue in paraffin blocks was sectioned into five 10 µm thick sections, mounted on Superfrost Plus slides, and air-dried overnight. After paraffin removal and rehydration of the tissue sections in phosphate-buffered saline for 48 hours, the tissue was transferred to an Eppendorf tube, and the fluid was completely discarded by centrifugation. Total DNA (genomic and plasmid DNA) was isolated by isopropanol precipitation. In short, tissues were lysed with proteinase K in TES buffer (50 mM Tris-HCl, 100 mM EDTA, 100 mM NaCl, 1% SDS, pH 8.0) for 5 hours at 56 °C. After the addition of 6 M NaCl, the solutions were vortexed and centrifuged for 5 min to obtain a clear solution, which was then further treated with isopropanol. The genomic DNA was precipitated by centrifugation (15 min; 13,000 rpm), washed with 70% ethanol, air-dried, and eluted in water.

Gene expression analysis was performed using polymerase chain reaction with Taq polymerase (Biozym #331610) and the following primers: Spike protein sequence 1 (221bp), fw CCTACACCAACAGCTTTACC, rev GATGTTGGACTTCTCGGTGC, Spike protein sequence 2 (212bp), fw CGCTTACTATGTGGGCTACC, rev TGGTGATATTGGGGAACCGC, Replication origin sequence (105bp), fw CTACATACCTCGCTCTGCTAATC, rev GCGCCTTATCCGGTAACTATC

A vial containing batch GH9715 was used as a positive control, as plasmid DNA from this batch was already isolated and sequenced and confirmed to refer to the plasmid sequence BNT162b2 (GenBank PP544445.1, GenBank PP544446.1, GenBank MQ287666.1 representing sequence 16 from patent WO2021214204). The PCR reactions were loaded onto a 2% agarose gel with ethidium bromide.

Immunohistochemistry: Formalin-fixed tissues were routinely processed, and paraffin-embedded tissues were cut into 5 μm sections and stained with marker S-100 (Medac, clone 4C4.9 #Z2055MT) for histopathological examination. Nuclei were counterstained with haematoxylin, and the images were acquired by a light microscope (Leica, Germany).
